# Coles' Deformities of the Mouth

**Published:** 1881-12

**Authors:** 


### BIBLIOGRAPHICAL.

*Deformities of the Mouth,* Congenital and Acquired, with their Mechanical Treatment. By Oakley Coles, of London, Lecturer on Dental Surgery and Deformities of the Mouth, in the National Dental College, etc., etc. Third edition. Publisher: Presley Blakiston, Philadelphia, 1881.

This work is illustrated by eighty-three wood engravings and ninety-six drawings in stone, and shows a careful revision by the author, having been entirely re-written, three new chapters added and the cuts increased. Although not so volumnious as Dr. Kingsley's work on the same subject, it is a valuable contribution to dental literature, and will prove serviceable to the dental profession. The greater portion of the volume is devoted to the consideration of Cleft Palate, congenital and acquired.

